# Development of a P2X1-eYFP receptor knock-in mouse to track receptors in real time

**DOI:** 10.1007/s11302-019-09666-1

**Published:** 2019-07-08

**Authors:** Martyn P. Mahaut Smith, Richard J. Evans, Catherine Vial

**Affiliations:** Department of Molecular and Cell Biology, University of Leicester, Leicester, LE1 7RH UK

**Keywords:** ATP, Megakaryocytes, P2X1-eYFP, Ion channels, Platelets, Smooth muscle, P2X1

## Abstract

A P2X1-eYFP knock-in mouse was generated to study receptor expression and mobility in smooth muscle and blood cells. eYFP was added to the C-terminus of the P2X1R and replaced the native P2X1R. Fluorescence corresponding to P2X1-eYFPR was detected in urinary bladder smooth muscle, platelets and megakaryocytes. ATP-evoked currents from wild type and P2X1-eYFP isolated urinary bladder smooth muscle cells had the same peak current amplitude and time-course showing that the eYFP addition had no obvious effect on properties. Fluorescence recovery after photobleaching (FRAP) in bladder smooth muscle cells demonstrated that surface P2X1Rs are mobile and their movement is reduced following cholesterol depletion. Compared to the platelet and megakaryocyte, P2X1-eYFP fluorescence was negligible in red blood cells and the majority of smaller marrow cells. The spatial pattern of P2X1-eYFP fluorescence in the megakaryocyte along with FRAP assessment of mobility suggested that P2X1Rs are expressed extensively throughout the membrane invagination system of this cell type. The current study highlights that the spatiotemporal properties of P2X1R expression can be monitored in real time in smooth muscle cells and megakaryocytes/platelets using the eYFP knock-in mouse model.

**Electronic supplementary material:**

The online version of this article (10.1007/s11302-019-09666-1) contains supplementary material, which is available to authorized users.

## Introduction

ATP is co-released with classical neurotransmitters and acts at P2X1 receptors (P2X1Rs) to mediate neurogenic contraction of arteries, urinary bladder and the vas deferens (1–3). In addition, extracellular ATP levels can rise in the circulation by regulated release from blood cells or as the result of tissue damage and activate P2X1Rs on platelets/megakaryocytes, neutrophils, macrophages, eosinophils and mast cells (4–8).

ATP opens the P2X1R channel resulting in an inward depolarizing current that decays during the continued presence of agonist (1–2 s) through a process called desensitisation. Receptor mobility has been suggested to contribute to the ability of a cell to respond to repeated applications of ATP (9). In order to visualise native P2X1 receptors in real time in vivo and ex vivo within primary cells, we have generated a P2X1-eYFP knock-in mouse. We have used this new mouse to assess the expression, localisation and mobility of P2X1Rs in acutely isolated smooth muscle cells, megakaryocytes and platelets.

## Materials and methods

The mouse P2X1R gene was linked in frame at the end of exon 12 to the enhanced yellow fluorescent protein (eYFP) via a 18 bp linker (5′-GATCCACCGGTCGCCACC-3′ corresponding to the amino acid sequence DPPVAT) in order to obtain the protein chimera P2X1-eYFP with the downstream neomycin cassette flanked by CRE-LoxP. Embryonic stem cells incorporating the P2X1-eYFP construct were selected by neomycin resistance and screened by Southern blot for correct homologous recombination of the P2X1R with P2X1-eYFP. Mice were then generated on a C57/Bl6 background and crossed with an MF1 CRE mice to remove the neomycin cassette (determined by PCR). Mice resulting from back crossing to MF1 of at least four generations were used in this study.

Single urinary bladder smooth muscle cells were isolated following enzymatic treatment and bathed in physiological solution containing (in millimolar): NaCl 150, KCl 2.5, HEPES 10, CaCl_2_ and MgCl_2_ 1 (pH adjusted to 7.3 with NaOH). Whole cell recordings were made with patch electrodes filled with a solution containing (in millimolar): Kgluconate 140, NaCl 5, HEPES 10, EGTA 9 (pH adjusted to 7.3 with KOH) using standard patch clamp recording conditions and ATP applied by U-tube as described previously (3). Tibial and femoral marrow was prepared as described previously (5). Blood was extracted from the vena cava into acid citrate dextrose anticoagulant under terminal anaesthesia in accordance with current Home Office legislation. The blood was centrifuged for 3 min at 300×*g* and the upper platelet-enriched plasma layer removed. This was diluted 1:100 into nominally Ca^2+^-free saline (10) for imaging.

P2X1-eYFP receptor distribution was imaged using a 60 × oil immersion 1.35 NA UPLSAPO objective lens on an Olympus IX81 microscope equipped with a FV1000 confocal laser scanning module (Olympus, UK). The dye was excited at 515 nm and the emission bandwidth was 530–630 nm. Receptor mobility was measured using fluorescence recovery after photobleach (FRAP) (9) and images were collected at 2 s intervals. After 20 baseline control images, the region of interest was subjected to a 1 s photobleach using high intensity 515 nm illumination (photobleach ~ 90%). Images were then collected for a further 260 s after photobleaching. Averaged intensities of bleached and background regions were recorded for each time-point. Fluorescence signals for the photobleached area were background subtracted and the percentage recovery relative to the initial fluorescence calculated.

Data are plotted as mean ± SEM and unpaired *t* tests were used for statistical analysis.

### Results

#### Detection of the P2X1-eYFP knock-in

**Fig. 1:**
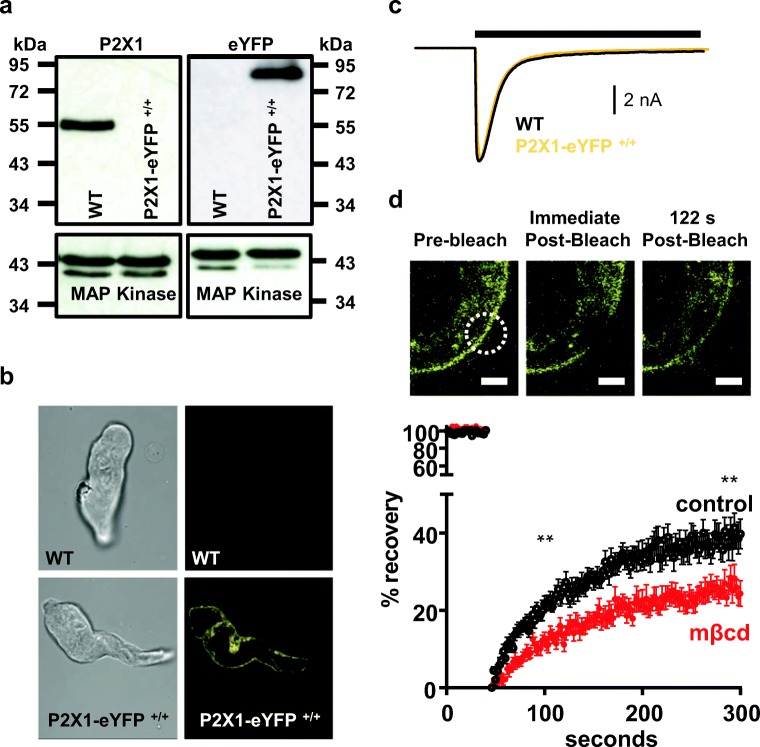
Properties of P2X1-eYFP receptors expressed in mouse urinary bladder. **a** Western blot of urinary bladder samples showing a band at ~ 55 kDa for the WT P2X1R that is absent in samples from the P2X1-eYFP +/+ mouse. In contrast the anti-GFP antibody detects a band of ~ 85 kDa from the P2X1-eYFP +/+ mouse that is absent from the P2X1R WT. Equivalent loading of WT and P2X1-eYFP +/+ samples is shown with the P42/44 MAP kinase antibody. **b** Brightfield (left) and fluorescent images of isolated urinary bladder smooth muscle cells from WT and P2X1-eYFP +/+ mice. **c** Patch clamp recordings of ATP (100 μM, 3 s application indicated by bar) evoked currents from WT and P2X1-eYFP +/+ acutely dissociated smooth muscle cells (holding potential − 60 mV). **d** P2X1-eYFP mobility was estimated by fluorescence recovery after photobleaching (FRAP). Top panel shows representative images of a peripheral area of the cell before, immediately after and 122 s after photobleaching the region indicated by the circle. Scale bars 2 μm. Lower panel shows mean data ± SEM of the percentage recovery of fluorescence to pre-bleaching levels. Black traces correspond to control conditions and red after 30 min treatment with methyl-β-cyclodextrin (mβcd) to deplete membrane cholesterol. **p < 0.01

Primers that detected the P2X1-eYFP construct (and the absence of cre) were used to screen mice and select those that were either homo- or heterozygous for P2X1-eYFP (data not shown). Confirmation of the correct genotype was obtained by Western blotting (Fig. 1a). For wild type (WT) mouse bladder, a ~ 55 kDa band was detected with the anti-P2X1R antibody generated against the C-terminus, consistent with previous work (11). However, no band at 55 kDa was detected for the homozygous P2X1-eYFP sample (even though there was equivalent protein loading shown by the p44/42 MAP kinase control) demonstrating that no WT P2X1R could be detected (for mice that were heterozygous for P2X1-eYFP, a 55 kDa product was detected—data not shown, that supports that the antibody can be used to detect WT receptor when the P2X1-eYFP is also present). The lack of P2X1R in the homozygous P2X1-eYFP sample is consistent with the addition of eYFP masking the C-terminal P2X1R epitope. To confirm P2X1-eYFP expression, we blotted the same samples with an anti-GFP antibody. The anti-GFP antibody detected bands at ~ 85 kDa for the P2X1-eYFP hetero- and homozygotes. These results are consistent with the P2X1-eYFP protein (predicted molecular weight ~ 85 kDa, ~ 55 kDa for P2X1 plus ~ 30 kDa for eYFP). For WT mice, no such band was detected.

#### Properties of P2X1-eYFP receptors in the bladder are indistinguishable from WT P2X1Rs

Fluorescence corresponding to eYFP could be detected from acutely dissociated bladder smooth muscle cells from P2X1-eYFP mice but not from WT controls (Fig. 1b). The addition of eYFP to the P2X1R carboxyl terminus had no effect on the peak current amplitude of ATP (100 μM, a maximal concentration) evoked currents in dissociated bladder cells (5242 ± 1245 and 5475 ± 1650 pA for WT and P2X1-eYFP, respectively, *n* = 8,7). In addition, the time-course of current decay during the continued application was indistinguishable between WT and P2X1-eYFP expressing cells (Fig. 1c). These results highlight that the eYFP has no effect on the properties of the P2X1R consistent with previous studies adding GFP (9). They show that the P2X1-eYFP receptor is expressed at normal levels and can be used to track in real time the location of the receptor.

#### Measurement of P2X1-eYFP mobility using FRAP

The mobility of proteins at the cell membrane can be measured using FRAP. For photobleaching, a 6 μm^2^ circle was selected at the surface of the bladder smooth muscle cell. This area was then photobleached with high intensity 515 nm light for 1 s that caused a ~ 90% decrease in fluorescence within the selected region. There was ~ 40% recovery of fluorescence in a 260 sec period, and this could be well fit with a single exponent with a time constant of 114 ± 13 s (*n* = 14). This recovery is ~ 2-fold slower than that reported previously for the P2X1-GFP receptor expressed in HEK293 cells (9) and suggests that in the native environment, there are additional factors, e.g. native protein interactions, that slow movement.

Cholesterol depletion is known to regulate P2XRs (for review, see (12)). For P2X1Rs, cholesterol depletion inhibits currents in recombinant and native systems (11). It has been shown that cholesterol depletion affects mobility in the membrane of a range of proteins (11). We therefore tested whether cholesterol depletion with methyl-β-cyclodextrin (1 mM for 30 mins) had an effect on the P2X1-eYFP FRAP profile to examine whether the tagged receptor displayed similar interactions with lipid rafts. Following cholesterol depletion there was a significant reduction in the amount of recovery (Fig. 1d) following photobleaching. This demonstrates that cholesterol regulates the mobility of the P2X1R in the membrane in native smooth muscle.

#### P2X1R-eYFP localisation on megakaryocytes and platelets

**Fig. 2:**
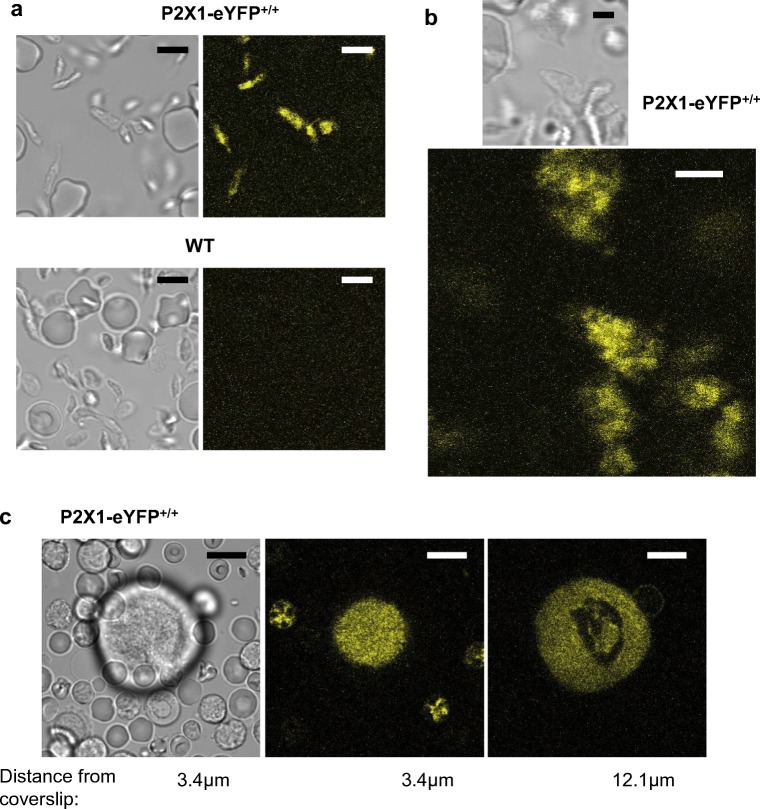
Expression of P2X1-eYFP receptors in platelets and megakaryocytes. Images collected by confocal microscopy from the platelet-enriched fraction of blood (**a**, **b**) or from dispersed marrow cells (**c**). Samples are from P2X1-eYFP mice (**a**, upper images, **b** and **c**) or a wild type control mouse (**a**, lower images). The fluorescence images have been pseudocoloured yellow. Images in **c** are from a z-stack of 88 images (for full movie see the supplementary video). Scale bars **a** 5 μm, **b** 2 μm and **c** 10 μm. Confocal thickness = 0.52 μm

Fluorescence imaging also demonstrated robust expression of P2X1-eYFPRs in platelets (Fig. 2a). The platelet-enriched plasma preparation contained a significant number of erythrocytes, which in contrast to platelets displayed negligible levels of eYFP. At high magnification, fluorescence could be observed throughout the platelet, although it was not possible to distinguish any pattern consistent with expression on the open canalicular system in addition to the peripheral surface membrane (Fig. 2b). The platelets were not adhered to the glass coverslip in order to avoid contact-dependent activation, and thus substantial cell movement prevented use of FRAP to assess receptor mobility.

P2X1-eYFP was also strongly expressed in megakaryocytes, with uniform fluorescence evident throughout the internal area with the exception of a region consistent with the polyploidic nucleus (Fig. 2c and supplementary movie of a z-series of 88 images). This suggests that the P2X1-eYFPR is present throughout the surface-connected demarcation membrane system (DMS) (13). Fluorescence was also detected in a small number of smaller marrow cells. Unlike platelets, the megakaryocytes settled on the coverslip without activation and thus P2X1-eYFP mobility could be assessed. Following a 1 s photobleach of eYFP within a cellular region corresponding to the DMS, ≈ 33% recovery of fluorescence was observed after 260 s. It was not possible to sufficiently separate fluorescence at the megakaryocyte cell surface membrane from the DMS. However, in smooth muscle, the recovery of internal receptors was < 10% (i.e. less than a quarter of the value observed for surface receptors). Presumably, this reflects the smaller compartments within which organellar P2X1 receptors are expressed compared to the surface membrane. The recovery rate for P2X1 receptors within the extranuclear volume of the MK was closer to that observed in the plasma membrane than in the organelles of smooth muscle and thus provides further evidence that P2X1 receptors are expressed throughout the DMS of the megakaryocyte.

## Discussion

The present study shows that a P2X1-eYFP knock-in mouse is viable and can be used to map the receptor expression in real time. P2X1-eYFP fluorescence was detected in the smooth muscle of the bladder (and arteries, data not shown) as well as platelets and megakaryocytes. There was no obvious expression over background levels in the central nervous system. These results are consistent with previous reports (14), and combined with the lack of effect of eYFP addition on P2X1R currents in the bladder smooth muscle, demonstrates that the knock-in mouse provides a useful resource for the real-time study of P2X1Rs in native tissues.

In the present study, FRAP measurements demonstrate that P2X1Rs are highly mobile within the cell membrane in native tissues, as previously shown for expression systems (9). Such mobility is important since it could replace desensitised receptors at an active site with “fresh” receptors from outside of the activated region. Within smooth muscle, localised activation of P2X1Rs following ATP release from autonomic nerves gives rise to P2X1R-mediated transient depolarizations called excitatory junction potentials (EJPs) (2). The EJPs facilitate to a sustained level within seconds (2) even though individual P2X1Rs desensitise in < 2 s and require ~ 3–5 min to recover (15). This apparent paradox has previously been explained by two factors: (1) transmitter release from individual varicosities innervating the muscle is intermittent (16) and (2) smooth muscle cells are electrically coupled and act as a functional syncytium. Therefore, the EJP corresponds to the summation of P2X1R activation at discrete sites distributed throughout the multicellular smooth muscle. The FRAP studies reported here within native tissue, and previously in heterologous expression systems (9), suggest that P2X1-eYFP receptor mobility/turnover is also likely to contribute to the steady-state neurogenic EJP in smooth muscle by providing a mechanism to replace P2X1Rs exposed to ATP adjacent to a nerve varicosity.

The present work also re-confirms that P2X1Rs are expressed in the platelet and megakaryocyte (17). The endogenous level of the tagged protein is sufficient to allow live cell fluorescence imaging and thus the transgenic model provides a tool for future in vivo studies of P2X1 receptor movements during megakaryopoiesis, thrombopoiesis and platelet functional responses. The spatial pattern of eYFP fluorescence along with FRAP measurements suggest that the receptor is extensively distributed throughout the DMS of the megakaryocyte. Super-resolution microscopy of the P2X1-eYFP alongside a fluorescent label of the DMS (18) may also reveal further complexities such as how the receptor is organised during the formation of proplatelets and subsequent release of platelets (19). This complex plasma membrane invagination system serves as a reservoir for platelet production. It is therefore likely that the P2X1 receptor is also resident within the platelet surface-connected open canalicular system. Further work using super-resolution imaging of static, non-activated platelets from the P2X1-eYFP mouse is required to confirm this and also whether the receptor is further compartmentalised to microdomains as previously proposed (17). This restricted distribution may result from known interactions with the cytoskeleton (20) and explain the ability of P2X1Rs to efficiently couple to functional responses through local activation of downstream Ca^2+^-dependent signalling events (17). The P2X1-eYFP mouse also provides a novel tool for assessing possible receptor distribution during platelet shape change and during interactions with other blood cells, particularly neutrophils (4). In addition to megakaryocytes, P2X1-eYFP was also detected in a small number of small marrow cells, although their haematopoietic subtype was not investigated. The P2X1-eYFP mouse now provides a new tool to examine expression of this Ca^2+^-permeable ion channel within different lineages of myeloid cells and its possible contribution to signalling within the complex environment of the marrow.

In summary, the present study reports a P2X1-eYFP-expressing mouse that has permitted the spatial distribution of the ion channel to be studied for the first time in native smooth muscle, megakaryocytes and platelets. This new model will be useful for the quantification of P2X1 distribution and mobility under a range of physiological and pathophysiological conditions both ex vivo and in vivo.

## Electronic supplementary material

ESM 1 (MP4 4874 kb)

ESM 2 (DOC 22 kb)
