# Supplementary material for: Development of a P2X1-eYFP receptor knock-in mouse to track receptors in real time
Source: Purinergic Signal. 2019 Jul 8;15(3):397–402. doi: 10.1007/s11302-019-09666-1 (PMC6736900; doi:10.1007/s11302-019-09666-1)
Supplement: Supplementary file 2 — (DOC 22 kb) [file 11302_2019_9666_MOESM2_ESM.doc]

**Supplementary Movie**

Confocal fluorescence image z-series through marrow cells isolated from a P2X1-eYFP^+/+^ mouse. Slice thickness 0.52 μm, interval 0.42 μm, total 88 slices. Right panel: eYFP fluorescence (515 nm excitation, 530-630nm emission); left panel: transmitted light signal. Acquired on an Olympus IX81 inverted microscope equipped with a FV1000 confocal module (Olympus, UK) using a 60x 1.3 NA UPLSAPO objective. Dimension of image: 56 μm x 56 μm.
